# Taxonomic Diversity and Antimicrobial Potential of Thermophilic Bacteria from Two Extreme Algerian Hot Springs

**DOI:** 10.3390/microorganisms13061425

**Published:** 2025-06-19

**Authors:** Marwa Aireche, Mohamed Merzoug, Amaria Ilhem Hammadi, Zohra Yasmine Zater, Keltoum Bendida, Chaimaa Naila Brakna, Meryem Berrazeg, Ahmed Yassine Aireche, Yasmine Saidi, Svetoslav Dimitrov Todorov, Dallel Arabet, Djamal Saidi

**Affiliations:** 1 Higher School of Biological Sciences of Oran, BP 1042 Saim Mohamed, Cité Emir Abdelkader (EX-INESSMO), Oran 31000, Algeria; marwa.ar231@gmail.com (M.A.); hammadiamaria267@gmail.com (A.I.H.); keltoumbendida2001@gmail.com (K.B.); chaimaa1012@gmail.com (C.N.B.); yasmine.saidi@gmail.com (Y.S.); djamsaidi@gmail.com (D.S.); 2 Laboratory of Microorganisms Biology and Biotechnology, University of Oran 1 Ahmed Ben Bella, Oran 31000, Algeria; yassminezater93@gmail.com; 3 Biotechnology Laboratory for Food and Energy Security, University of Oran 1 Ahmed Ben Bella, Oran 31000, Algeria; berrazegmeryem@gmail.com; 4 Computer Science Department, University of Abu Bekr Belkaid, Tlemcen 13000, Algeria; yassinetakiko@gmail.com; 5 ProBacLab, Laboratório de Microbiologia de Alimentos, Departamento de Alimentos e Nutrição Experimental, Food Research Center, Faculdade de Ciências Farmacêuticas, Universidade de São Paulo, São Paulo 05508-000, SP, Brazil; 6 Department of General Hygiene, I.M. Sechenov First Moscow State Medical University, Trubetskaya St., Bldg. 8/2, Moscow 119435, Russia; 7 Laboratory of Microbiological Engineering and Applications, Faculty of Natural and Life Sciences, University of Constantine 1, Constantine 25000, Algeria; dallelarabet@yahoo.fr

**Keywords:** thermophiles, extreme environments, MALDI-TOF MS, rep-PCR fingerprinting, 16S rRNA, antimicrobial peptides (AMPs), biotechnological applications

## Abstract

This study investigated thermophilic bacterial communities from two Algerian hot springs: Hammam Debagh (94–98 °C), recognized as the second hottest spring in the world, and Hammam Bouhadjar (61–72 °C), one of the hottest in northwest Algeria. Thirty isolates were obtained, able to grow between 45 °C and 80 °C, tolerating pH 5.0–12.0 and NaCl concentrations up to 3%. Colonies displayed diverse morphologies, from circular and smooth to star-shaped and Saturn-like forms. All isolates were characterized as Gram-positive, catalase-positive rods or filamentous bacteria. Identification by MALDI-TOF, rep-PCR and 16S rRNA sequencing classified them mainly within *Bacillus*, *Brevibacillus*, *Aneurinibacillus*, *Geobacillus*, and *Aeribacillus*, with *Geobacillus* predominating. Rep-PCR provided higher resolution, revealing intra-species diversity overlooked by MALDI-TOF MS and 16S rRNA. A subset of six isolates, mainly *Geobacillus* spp., was selected based on phenotypic and genotypic diversity and tested for antimicrobial activity against thermophilic target isolates from the same hot spring environments. Strong inhibition zones (~24 mm) were observed, with *Geobacillus thermoleovorans* B8 displaying the highest activity. Optimization on Modified Nutrient Agar medium with Gelrite enhanced antimicrobial production and inhibition clarity. These findings highlight the ecological and biotechnological significance of thermophilic bacteria from Algerian geothermal ecosystems. While this study focused on microbial interactions within thermophilic communities, the promising inhibitory profiles reported here provide a foundation for future research targeting foodborne and antibiotic-resistant pathogens, as part of broader efforts in biopreservation and sustainable antimicrobial development.

## 1. Introduction

Extremophiles have developed specialized mechanisms to survive extreme conditions, with their biology shaped by harsh physical and chemical factors [1,2]. Thermophilic bacteria, thriving in high-temperature environments like hot springs and geothermal vents, hold great promise as a source of novel bioactive molecules [3,4,5]. Their unique biochemical traits, including thermostable enzymes and bioactive metabolites, make them promising candidates for mapping new antimicrobial peptides (AMPs), including bacteriocins, which remain effective at high temperatures [6,7]. Unlike conventional antibiotics, these thermostable compounds resist very high temperatures and show strong activity against pathogens in clinical settings, as well as thermoresistant microorganisms in industrial, agricultural, and food processing applications [8,9,10]. Bacteriocins, with their targeted activity, are particularly useful for controlling specific microbes while preserving beneficial microbiota. Their applications include food preservation, crop protection, and combating multidrug-resistant pathogens [11,12,13].

The extreme physicochemical conditions of hot springs, such as elevated temperatures, variations in pH, and fluctuations in oxygen levels, promote the growth of specialized microbial communities, making these sites valuable sources of new bioactive compounds with significant biotechnological potential [14,15].

Algeria hosts over 240 geothermal springs, yet their microbial diversity remains largely unexplored [16]. Among them, Hammam Debagh, with temperatures reaching 98 °C, is the hottest hot spring in Africa and the second hottest worldwide [17,18]. Hammam Bouhadjar, with temperatures ranging from 61 °C to 72 °C, ranks among the hottest springs in northwestern Algeria [19,20]. These extreme environments are ideal for isolating thermophilic and extremophilic microorganisms with unique metabolic traits [21]. Despite their potential, these sites have attracted limited scientific investigation. Exploring their microbial resources could lead to major advances in industrial, agricultural, and pharmaceutical applications [22,23,24].

For effective exploitation of microbial communities, accurate and integrative identification methods are essential. Traditional phenotypic techniques are limited, especially for closely related species. Thus, proteomic and molecular tools have become central to microbial taxonomy and functional screening. Moreover, microbial communities in extreme environmental conditions are facing additional challenges, since most of the microbial research approaches need to be adapted to the specific conditions, where not only high temperatures are a specific factor, but salinity, specific non-organic salts, and pH are often in ranges considered extreme as well [25,26].

A powerful tool in bacterial identification is Matrix-Assisted Laser Desorption/Ionization-Time of Flight (MALDI-TOF) mass spectrometry, which enables rapid species-level identification by comparing protein profiles to reference databases. Its speed and precision make it appropriate for high-throughput screening in both environmental and clinical microbiology, though its reliability hinges on database completeness and accuracy [27,28].

Complementing this, 16S rRNA gene sequencing remains a molecular gold standard for phylogenetic identification. By targeting conserved regions of the 16S gene, this method enables robust species-level classification and offers deep insights into microbial diversity and evolutionary relationships [29].

Combined with rep-PCR (Repetitive Extragenic Palindromic PCR), it further enhances strain-level resolution by generating genomic fingerprints based on repetitive DNA elements. This technique provides valuable data on genetic diversity, enabling population structure analysis and fine-scale discrimination among closely related isolates [30].

This study aimed to isolate and characterize moderate and extreme thermophilic bacteria from two prominent geothermal springs in Algeria, Hammam Bouhadjar and Hammam Debagh, which are renowned for their extreme temperatures. Hammam Bouhadjar is known for its high temperatures in northwest Algeria, while Hammam Debagh holds the distinction of being the second hottest spring globally. Although thermophilic bacteria are known for their biotechnological significance, the genetic diversity of these microorganisms in such extreme environments remains largely unexplored. To address this knowledge gap, a polyphasic approach was adopted, combining MALDI-TOF mass spectrometry, 16S rRNA gene sequencing, and genetic fingerprinting using (GTG)_5_, BOX, and ERIC primers. This comprehensive methodology offers detailed insights into the taxonomic and genetic makeup of these extremophiles, supporting the evaluation of their potential applications in medicine, industry, and biotechnology.

## 2. Materials and Methods

### 2.1. Environmental Sampling and Site Description

Between June 2023 and February 2024, water samples were collected from two Algerian hot springs: Hammam Debagh (94–98 °C) in the northern region (approximately 36°27′36.3″ N, 7°16′11.7″ E) and Hammam Bouhadjar (61–72 °C) in the northwest region (approximately 35°22′04.6″ N, 0°58′10.4″ W). Thermophilic microorganisms’ viability was prioritized during all stages of sample collection, transportation, and analysis. To minimize thermal shock and maintain the integrity of the thermophilic microorganisms, environmental samples were transported in insulated containers that preserved the thermal conditions of the source environment. Upon arrival at the laboratory, the samples were divided into two aliquots. Each aliquot was stored under two different conditions: one at room temperature and the other at 4 °C. Both storage conditions lasted less than 12 h before the microbiological processing was conducted [31,32]. These samples were then used for the isolation and characterization of thermophilic microbial communities.

### 2.2. Isolation of Thermophilic Bacteria

Two methods were used to isolate thermophilic bacteria: liquid enrichment (20 mL of water sample inoculated into 100 mL of autoclaved concentrated liquid medium) and membrane filtration (1 L of sample filtered through 0.22 µm membranes, then transferred onto agar plates). A range of culture media was employed to support the growth of diverse thermophilic isolates, including Nutrient Broth (NB) and Nutrient Agar (NA; Sigma-Aldrich, Saint Louis, MO, USA), Thermophilic Agar (TA) (g/L, starch 10 g, yeast extract 2 g, peptone 1 g, glucose 2 g, NaCl_2_ g, KNO_2_ 2 g, K_2_HPO_4_ 0.5 g, MgSO_4_.7H_2_O 0.5 g, CaCO_3_ 1 g, agar 20 g, pH 7.0), Modified Nutrient Agar (MNA) (glucose 2 g, peptone 15 g, NaCl 6 g, yeast extract 5 g, tryptone 10 g, agar 20 g, pH 7.0), Tryptone Soy Agar (TSA; MP Biomedicals, Solon, OH, USA), and Czapek-Dox Agar (CDA) (g/L, Saccharose 30 g, NaNO_3_ 2 g, K_2_HPO_4_ 1 g, MgSO_4_ 0.5 g, KCl 0.5 g, FeSO_4_.7H_2_O 0.01 g, agar 20 g, pH 7). The plates were incubated at 60 °C for 2–4 days. Colonies were selected based on morphological characteristics, purified by successive streaking until single colonies were obtained, and stored at 4 °C for short-term use. For long-term preservation, cultures were stored in 20% glycerol at –20 °C [33].

### 2.3. Phenotypic and Physiological Characterization of Isolates

Morphological characterization was performed by examining the shape, size, and spore formation of the bacterium under a compound microscope (Optika Microscopes, Bergamo, Italy). Cell morphology was examined through Gram staining, and catalase activity was tested [34,35]. For precise determination of optimal growth conditions, the temperature range for growth was assessed by incubating cultures from 40 °C to 80 °C with 5 °C increments. The effect of pH on bacterial growth was evaluated across a pH range of 4.0 to 12.0, while the impact of NaCl on bacterial growth was studied in Nutrient Broth (Sigma-Aldrich) containing 1%, 2%, 3%, and 5% NaCl (*w*/*v*). The bacterial isolates from the geothermal sites were routinely maintained at 4 °C after incubation on NA (Sigma-Aldrich) for 24–48 h between 60 °C and 75 °C.

### 2.4. Proteomic Identification Using MALDI-TOF Mass Spectrometry

Bacterial isolates were identified using Matrix-Assisted Laser Desorption/Ionization-Time of Flight System, MALDI-TOF Biotyper^®^ sirius with a Microflex LT system (Bruker Daltonics, Bremen, Germany). Fresh bacterial colonies were directly spotted onto the MALDI target plate, treated with 1 µL of 70% formic acid (Sigma-Aldrich) to facilitate Gram-positive cell lysis [36], and overlaid with 1 µL of α-cyano-4-hydroxycinnamic acid (HCCA; 10 mg/mL; Bruker Daltonics) matrix dissolved in Organic Solvent (OS; VWR International, LLC, Radnor, PA, USA). After air-drying, mass spectra were acquired using FlexControl software (version 3.4) (Bruker Daltonics) and analyzed with MBT Compass HT software (version 5.1) (Bruker Daltonics). Calibration was performed using the Bacterial Test Standard (BTS; Bruker Daltonics). Identification was based on spectral comparison with a reference database, with scores above 2.0 considered indicative of high-confidence matches. Each isolate was tested in triplicate, and the final identification was based on the average score of the replicates [37,38].

### 2.5. Molecular Identification

#### 2.5.1. Genomic DNA Extraction and Quality Assessment

Genomic DNA was extracted from freshly grown bacterial isolates cultivated in NB at 60 °C using the PureLink Genomic DNA Mini Kit (Thermo Scientific, Waltham, MA, USA), with 50 µL of lysozyme (40 mg/mL) to improve cell lysis [39]. The process followed the manufacturer’s protocol, including centrifugation and pellet recovery. DNA concentration and purity were assessed with a ScanDrop spectrophotometer (Analytik Jena, Jena, Germany). DNA integrity was verified by gel electrophoresis on a 0.8% agarose gel. Samples were stored at −20 °C for future use.

#### 2.5.2. Genomic Fingerprinting via rep-PCR ((GTG)_5_-PCR, BOX-PCR, ERIC-PCR)

Fingerprinting of 30 bacterial isolates was carried out using rep-PCR with previously described primers (Table 1), synthesized at the Genomics Technology Platform of the Higher School of Biological Sciences in Oran (Oran, Algeria). The protocol was modified with optimized reagent volumes based on the method of Adiguzel et al. [40]. Each 40 µL PCR reaction contained 3 µL of purified genomic DNA (~100 ng), 20 µL of DreamTaq PCR Master Mix (2×; Thermo Scientific), 1 µL of dimethyl sulfoxide (DMSO, 100%; New England Biolabs, UK), 1.75 µL of bovine serum albumin (BSA, 20 mg/mL, fraction V, 96–99% purity; Sigma-Aldrich), and 11.25 µL of nuclease-free water. For BOX and (GTG)_5_ reactions, 3 µL of primer (5 µM) was added, while ERIC reactions included 1.5 µL of each forward and reverse primer. A negative control (without DNA template) was included in each assay. PCR amplification was carried out on a SimpliAmp Thermal Cycler (Applied Biosystems, Foster City, CA, USA) using the same thermal cycling conditions, modified to include 35 amplification cycles. About 15 µL of the PCR products was mixed with 3 μL of 6× gel loading buffer and subjected to agarose gel electrophoresis (1.5% *w*/*v*) in 80 mL of 1% TBE (Tris–Borate–EDTA) buffer at 100 V for 120 min. The gel was stained with SYBR Safe DNA Gel Stain (Thermo Scientific). The amplified DNA product was visualized using the ChemiDoc Imaging System (Bio-Rad Laboratories, Hercules, CA, USA), with a GeneRuler Express DNA Ladder (Thermo Scientific) as a molecular size marker. Electrophoretic patterns were analyzed using GelJ software (version 2.0.0) to normalize migrations relative to the molecular weight marker [41]. Genetic distances were calculated using Dice similarity coefficients, and dendrograms were constructed using the UPGMA clustering method to assess the relationships among strains [42,43].

#### 2.5.3. 16S rRNA Gene Amplification

For molecular identification, the 16S rRNA gene was amplified using universal bacterial primers 16S Forward 27F and 16S Reverse 1495R (Table 1) [44,45], synthesized at the Genomics Technology Platform of the Higher School of Biological Sciences of Oran (Oran, Algeria). PCR reactions were prepared in a final volume of 30 µL, using DreamTaq PCR Master Mix (2×) (Thermo Scientific), 1 µL of purified genomic DNA (approximately 50 ng), 0.8 µL of each forward and reverse primer, and nuclease-free water. The amplification was performed on the SimpliAmp thermal cycler (Applied Biosystems). Thermal cycling conditions consisted of an initial denaturation at 95 °C for 10 min, followed by 35 cycles of denaturation at 94 °C for 45 s, annealing at 56 °C for 45 s, and extension at 72 °C for 1 min 30 s, with a final extension at 72 °C for 7 min to ensure complete elongation. The resulting amplicons (~1500 base pairs) were verified using 1% agarose gel electrophoresis in 1% TBE buffer, alongside the 1 kb Plus DNA Ladder (Thermo Scientific) for size verification.

#### 2.5.4. 16S rRNA Sequencing Analysis

Successfully amplified 16S rRNA gene fragments were purified using enzymatic cleanup with ExoSAP-IT PCR Product Cleanup Reagent (Applied Biosystems), following the manufacturer’s instructions to remove excess primers and unincorporated nucleotides [48]. Sanger sequencing was performed bidirectionally with the BigDye Terminator v3.1 Cycle Sequencing Kit (Applied Biosystems). The thermal cycling conditions included 25 cycles of denaturation at 96 °C for 10 s, annealing at 50 °C for 5 s, and extension at 60 °C for 4 min. Sequencing reaction products were purified using the DynaBeads Sequencing Clean-Up Kit (Thermo Scientific) to effectively remove excess dye terminators and contaminants. The purified products were eluted in Hi-Di Formamide (Thermo Scientific) and analyzed via capillary electrophoresis using the ABI 3500 Genetic Analyzer (Thermo Scientific) at the Genomics Technology Platform of the Higher School of Biological Sciences of Oran (Oran, Algeria). The 16S rRNA gene sequences of all 30 thermophilic isolates were cleaned and aligned, with the average consensus length obtained being 800 ± 20 bp when compared to their closest phylogenetic relatives.

#### 2.5.5. Phylogenetic Analysis

Chromatograms from Sanger sequencing were assessed for quality using SnapGene Viewer (v7.2.1), followed by sequence editing and alignment in BioEdit (v7.7.1) [49]. The edited sequences were compared to NCBI GenBank entries using BLASTn [50] to determine the closest taxonomic affiliations. The phylogeny was inferred using the Maximum Likelihood method and the 2-parameter model of nucleotide substitutions [51]. The tree with the highest log-likelihood (−10,637.87) is presented. The percentage of replicate trees in which the associated taxa clustered together (out of 500 replicates) is indicated next to the branches [52]. The initial tree for the heuristic search was chosen by selecting the one with the superior log-likelihood between a Neighbor-Joining (NJ) tree [53] and a Maximum Parsimony (MP) tree. The NJ tree was constructed using a pairwise distance matrix computed via the *p*-distance method [54]. The MP tree was derived from the shortest tree length obtained from 10 MP searches, each initiated with a randomly generated starting tree. Evolutionary rate differences among sites were modeled using a discrete Gamma distribution with 5 categories (+G, parameter = 2.6760), and 8.96% of sites were considered evolutionarily invariant (+I). The analysis encompassed 42 coding nucleotide sequences, including 1st, 2nd, and 3rd codon positions, as well as non-coding positions, resulting in a final dataset of 1718 positions. Evolutionary analyses were conducted using MEGA12 (v12.0.8) [55], utilizing up to 4 parallel computing threads. MUSCLE software [56], available within MEGA12, was utilized to generate reliable sequence alignments.

### 2.6. Screening for Antimicrobial Activity

The antimicrobial activity of six thermophilic isolates (B8, B70, B14, B16, G4, and G30) was tested against phylogenetically related thermophilic indicators (G10, B18, B9, G5, B15, and B70) isolated from the same site. The assay was performed using the agar well diffusion method with minor modifications based on Balouiri et al. [57]. Test isolates were cultured on Nutrient Agar (NA) at 60 °C for 24–48 h. Indicator isolates were grown under the same conditions and adjusted to a 0.5 McFarland standard (~1.5 × 10^8^ CFU/mL), then inoculated onto soft NA plates (0.75% agar) supplemented with 0.25% Gelrite (Serva Electrophoresis GmbH, Heidelberg, Germany) to enhance clarity and improve zone measurement [58]. Wells (6 mm diameter) were made using sterile pipette tips and filled with 100 µL of cell-free supernatants, prepared by centrifugation at 10,000× *g* for 15 min at 4 °C. Supernatant pH was adjusted to 6.5–7.0. Plates were pre-diffused at 4 °C for 2 h, then incubated at 60 °C for 24 h. Inhibition zones were measured in millimeters, with all tests conducted in triplicate. Non-inoculated media served as negative controls. The protease sensitivity of antimicrobial compounds was assessed by treating cell-free supernatants with proteinase K (1 mg/mL; Sigma-Aldrich) at 37 °C for 2 h, followed by enzyme inactivation at 100 °C for 3 min. Antimicrobial activity was then evaluated using the agar well diffusion method against the same indicator isolates, with untreated supernatants as controls [59].

### 2.7. Optimization of Antimicrobial Metabolite Production

The production of antimicrobial metabolites by thermophilic isolates was optimized by culturing the most bioactive isolates in various media, including Luria-Bertani (LB) (Merck), Mueller–Hinton (MH), Nutrient Broth (NB) (Sigma-Aldrich), and Modified Nutrient Broth (MNB). The pH of each medium was adjusted to 7.0 before autoclaving. Cultures were grown in 500 mL Erlenmeyer flasks containing 100 mL of medium, at 60 °C with agitation at 150 rpm. Incubation periods of 24, 48, and 72 h were used, after which the cultures were centrifuged at 10,000× *g* for 15 min. The supernatant was then collected for antimicrobial testing, and solid media with Gelrite (Serva) concentrations (0.25%, 0.5%, and 1%) were also used. Cultures of *Enterococcus faecium* were heat-killed (121 °C for 15 min) and then centrifuged at 10,000× *g* for 10 min. The resulting supernatant was filtered and inoculated with the inhibitory isolates’ cultures into Erlenmeyer flasks for co-cultivation to stimulate antimicrobial activity [60].

### 2.8. Data Processing and Statistical Analysis

All experiments were conducted in triplicate. Statistical analyses were performed using a one-way analysis of variance (ANOVA) to assess differences between experimental groups, with a significance threshold set at *p* < 0.001. Results are expressed as the mean ± standard deviation (SD). Data visualization and distribution analyses were conducted using Python (v3.13) with Hierarchical Cluster Analysis (HCA), employing the following libraries: NumPy (v2.3.0) for numerical computations [61], Pandas (v2.3.0) for data manipulation [62], Matplotlib (v3.10.3) [63] and Seaborn (v0.13.2) [64] for plotting, and SciPy (v1.15.3) [65] and Scikit-learn (v1.7.0) [66] for statistical analysis.

## 3. Results

### 3.1. Environmental Sampling and Bacterial Isolation

A total of 30 thermophilic bacterial isolates were successfully isolated and purified from water samples collected from two Algerian hot springs: 10 isolates from Hammam Debagh (labeled with “G”) and isolates from Hammam Bouhadjar (labeled with “B”). Among the six tested media, MNB, TA, and TSA yielded the highest number of isolates, indicating their suitability for supporting thermophile growth. The dual-storage protocol follows established practices in microbial ecology aimed at minimizing potential biases in microbial recovery due to temperature-induced shifts during short-term storage. After the short-term storage period, no significant differences were observed in the number or type of thermophilic isolates recovered from samples stored at room temperature and those stored at 4 °C. Since storage conditions did not influence microbial recovery or community composition, this comparison was not further discussed, as it did not affect the results or interpretation of the study.

### 3.2. Phenotypic Characterization and Optimum Physiological Parameters

**Figure 1:**
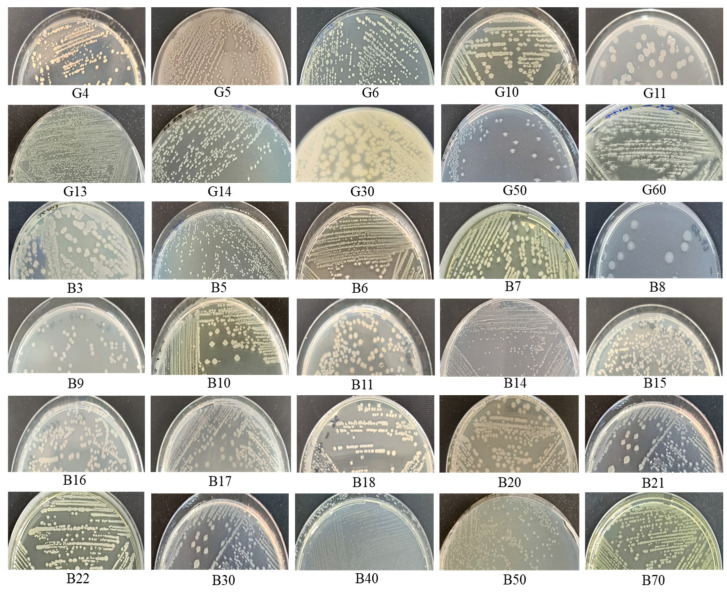
Macroscopic appearance and morphological diversity of thermophilic bacterial isolates (G and B isolates) grown on Nutrient Agar, originating from two Algerian hot springs: Hammam Debagh (G) and Hammam Bouhadjar (B).

Aerobic thermophilic bacteria were successfully isolated from hot spring samples and exhibited notable phenotypic diversity. Colonies on solid media showed a wide range of morphologies. Most were circular, with smooth or slightly wavy edges, and smooth to shiny surfaces, in colors ranging from off-white to pale cream (e.g., *Brevibacillus aydinogluensis* G4, *Brevibacillus aydinogluensis* G6, *Geobacillus proteiniphilus* B11, *Geobacillus kaustophilus* B14, *Geobacillus vulcani* B18, and *Geobacillus kaustophilus* B22). Some colonies were star-shaped with similar surface characteristics (*Aneurinibacillus thermoaerophilus* G60). Others presented distinctive appearances, including colonies with radial filaments and saturn-like colonies (e.g., *Geobacillus kaustophilus* G10, *Geobacillus stearothermophilus* G14, *Brevibacillus thermoruber* G50, *Geobacillus kaustophilus* B21, and *Geobacillus thermoleovorans* B30), and others have a dense and opaque central region, followed by a clearer area, then become dense and opaque at the edges (*Geobacillus proteiniphilus* G11, *G. thermoleovorans* B8) (Figure 1). All isolates were Gram-positive and catalase-positive, exhibiting rod or filament-shaped cells under microscopic observation. In terms of growth conditions, all isolates tolerated temperatures between 45 °C and 75 °C, with optimal growth observed at 70 °C (43.3% of isolates), 60 °C (23.3%), and 55 °C (33.4%). Notably, some isolates tolerated 80 °C, indicating high thermotolerance. pH tolerance ranged from 5.0 to 10.0, with optimal growth between pH 6.0 and 8.0. Isolates grew in up to 3% NaCl, with optimal growth at 1%, indicating moderate halotolerance.

### 3.3. MALDI-TOF MS Identification

The proteomic identification of the thermophilic bacterial isolates using MALDI-TOF MS revealed that most isolates were successfully assigned to known thermophilic genera, particularly *Geobacillus*, *Brevibacillus*, *Bacillus*, and *Aeribacillus*. All the isolates were analyzed, revealing eight distinct species. *G. kaustophilus* was the predominant species, with 14 isolates identified from Hammam Bouhadjar. Other species included *Geobacillus jurassicus*, *Bacillus licheniformis*, *Aeribacillus pallidus*, *Bacillus aydinogluensis*, *Geobacillus stearothermophilus*, *Geobacillus thermoleovorans*, and *Aneurinibacillus thermoaerophilus*. The majority of the MALDI-TOF scores were higher than 2.0, indicating reliable and confident species-level identification. However, some isolates, such as *G. proteiniphilus* G11, *B. thermoruber* G50, *Bacillus paralicheniformis* B3, *G. proteiniphilus* B17, and *G. thermoleovorans* B30, exhibited lower scores (around 1.7–1.9), requiring further molecular analysis (Table 2).

### 3.4. Comparative Analysis of BOX, ERIC, and (GTG)_5_-PCR Fingerprinting Profiles

**Figure 2:**
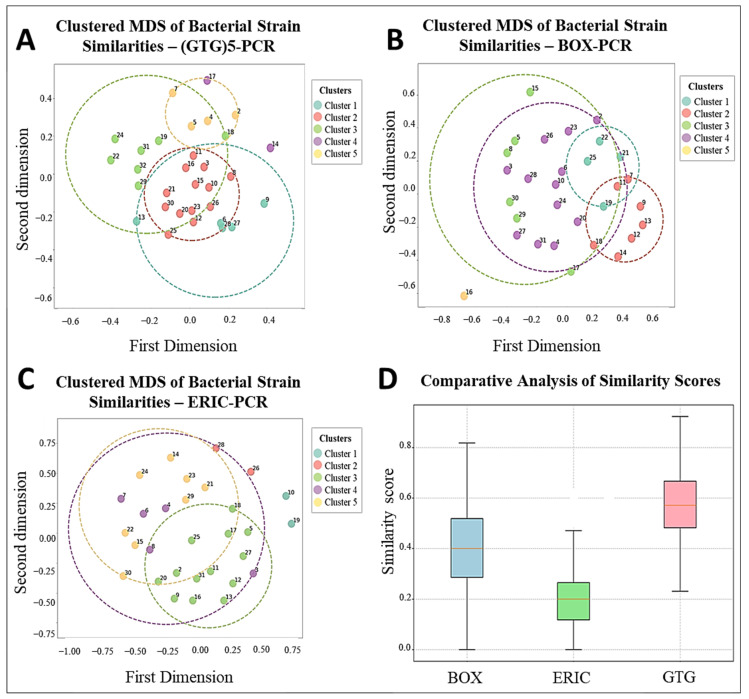
Comparative analysis of similarity index distributions and scores among thermophilic isolates assessed by (GTG)_5_-PCR, BOX-PCR, and ERIC-PCR techniques. Clustered multidimensional scaling (MDS) representations were generated to visualize bacterial strain similarities for each method: (GTG)_5_-PCR (**A**), BOX-PCR (**B**), and ERIC-PCR (**C**), with numbers representing unique isolates and colors indicating genetic clusters. A box plot (**D**) compares the similarity scores obtained across the three techniques, highlighting differences in variability, central tendency, and discriminatory resolution among the methods.

To differentiate the 30 purified thermophilic isolates, rep-PCR techniques including (GTG)_5_-PCR, BOX-PCR, and ERIC-PCR were employed. Banding profiles were analyzed using GelJ software (v2.0.0), and Dice similarity matrices were calculated and clustered using the UPGMA algorithm. Multidimensional Scaling (MDS) in Python (v3.13) was used for visual representation. Each method revealed five primary genetic clusters with distinct polymorphic profiles, as shown in Figure 2 (panels A, B, and C). Similarity indices ranged from ~40% to 95% across all methods, indicating substantial genetic diversity.

**Figure 3:**
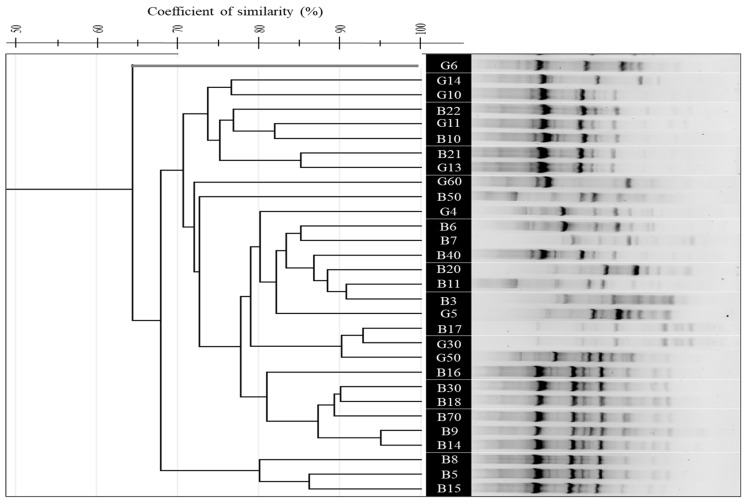
UPGMA dendrogram showing the genetic relatedness of 30 thermophilic bacterial isolates from Hammam Debagh and Hammam Bouhadjar hot springs based on (GTG)_5_-PCR fingerprinting profiles using (GTG)_5_ primer.

The (GTG)_5_-PCR profiles showed tight clustering, with Dice similarity coefficients ranging from 65% to 95%, indicating moderate to high genetic relatedness. The *G. kaustophilus* isolates B5, B10, and B70 formed compact clusters, while *Bacillus licheniformis* G30 and *B. paralicheniformis* B3 clustered together, suggesting limited interspecific resolution. Isolates from Hammam Debagh and Hammam Bouhadjar occasionally clustered together, indicating shared genomic features. (GTG)_5_-PCR profiles revealed significant diversity, particularly in the middle (for *B. licheniformis* G30 to *A. thermoaerophilus* G60) and lower sections, while the upper lanes were more homogeneous, with sharp, well-defined bands and minimal background noise (Figure 2A and Figure 3).

**Figure 4:**
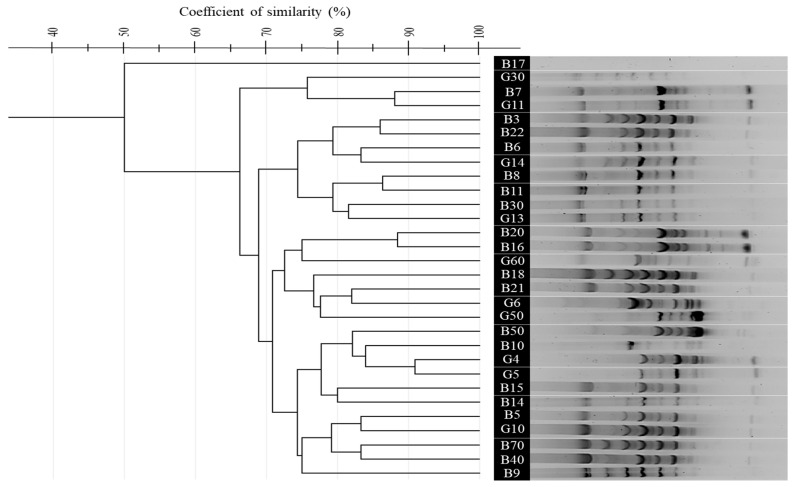
UPGMA dendrogram showing the genetic relatedness of 30 thermophilic bacterial isolates from Hammam Debagh and Hammam Bouhadjar hot springs based on BOX-PCR fingerprinting profiles using BOXA1R primer.

In contrast, BOX-PCR showed a broader spatial distribution in the MDS plot, with Dice similarity values ranging from 50% to 90%. Certain isolates, like *G. thermoleovorans* B30 and *B. licheniformis* G30, were outliers, indicating distinct genomic fingerprints. *A. pallidus* isolates (B40, B50) were clearly separated, while *B. aydinogluensis* (G4, G5) remained closely grouped. Several strains, including *Geobacillus stearothermophilus* G13, *G. stearothermophilus* G14, and *G. thermoleovorans* B30, consistently clustered together. The BOX-PCR gel displayed distinct banding patterns, with some strains (e.g., *G. kaustophilus* B9, *G. kaustophilus* B10, *G. kaustophilus* B15) showing unique profiles, while others (e.g., *G. kaustophilus* G10, *G. kaustophilus* B5, *G. kaustophilus* B14) were closely related (Figure 2B and Figure 4).

**Figure 5:**
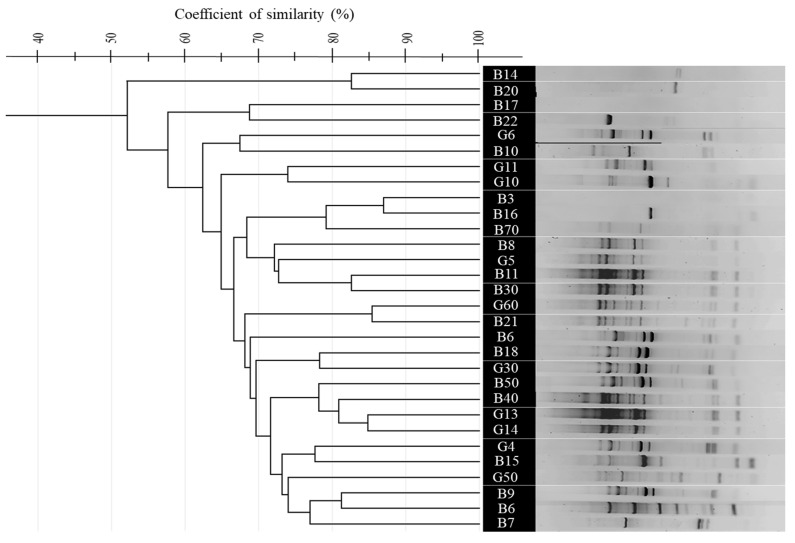
UPGMA dendrogram showing the genetic relatedness of 30 thermophilic bacterial isolates from Hammam Debagh and Hammam Bouhadjar hot springs based on ERIC-PCR fingerprinting profiles using ERIC 1R and ERIC 2 primers.

ERIC-PCR showed similarity indices from 52% to 85%, indicating lower resolution with broader clusters and less sub-structuring among *G. kaustophilus* strains. However, *G. stearothermophilus* (G13, G14) formed a distinct cluster (>90%), and *A. pallidus* isolates clustered tightly, differing from BOX-PCR results. The ERIC-PCR gel presented faint and variable bands, with some samples showing complex bands and others faint ones. Notably, lanes from *G. kaustophilus* B21 to *G. thermoleovorans* B8 clustered together, suggesting close relationships. Additionally, 9 isolates in (GTG)_5_-PCR, 11 in BOX-PCR, and 8 in ERIC-PCR showed unique banding patterns (Figure 2C and Figure 5). 

A boxplot comparison of similarity distributions confirmed that (GTG)_5_-PCR had the highest median similarity and strongest resolution among closely related strains. BOX-PCR displayed a wider score range, reflecting broader differentiation capacity. ERIC-PCR exhibited the lowest median similarity and a narrower distribution, reinforcing its limited discriminatory power but potential to detect macro-patterns and some unique outliers (Figure 2D). 

### 3.5. Sequencing Analysis and Phylogenetic Analysis

**Figure 6:**
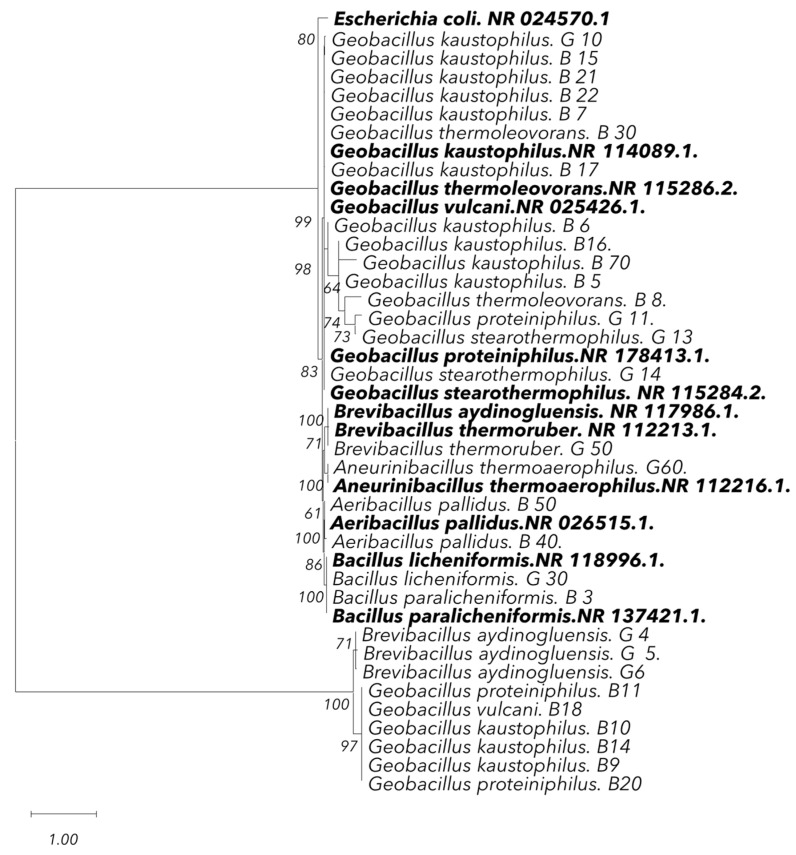
Phylogenetic tree based on 16S rRNA gene sequences of 30 thermophilic isolates (regular font) and 12 reference isolate strains (in bold font, along with their NCBI accession numbers), constructed using the Maximum Likelihood method [51] with MEGA12 software (v12.0.8) [55]. Evolutionary relationships were inferred under the 2-parameter nucleotide substitution model (+Γ, 5 categories; +I, 8.96% invariant sites). Bootstrap support values (500 replicates) [52] are shown along branches. Sequences were aligned using MUSCLE [56] to ensure high accuracy.

The molecular identification of the thermophilic bacterial isolates through 16S rRNA gene sequencing provided a highly accurate and detailed characterization. Analysis revealed the presence of 11 distinct species among the isolates. The dominant species was *G. kaustophilus*, confirmed in a large number of isolates, consistent with the proteomic findings. Additional species identified included *G. proteiniphilus*, *B. thermoruber*, *G. vulcani*, and *B. paralicheniformis*. Most isolates exhibited very high similarity rates (typically greater than 99%), and a few isolates, such as *G. kaustophilus* B15 (97.96%) and *G. kaustophilus* B16 (97.32%), showed slightly lower similarity percentages (Table 2). Moreover, significant phylogenetic clustering was revealed among the thermophilic isolates from the hot springs, particularly within the genus *Geobacillus*, with a specific focus on *G. kaustophilus*. The majority of the isolates from both hot springs clustered together, forming distinct clades with high bootstrap support (94%, 99%). This suggests a close genetic relationship among the isolates from the same thermal environment, further indicating that they may share similar ecological adaptations, particularly to high-temperature conditions (Figure 6). 

### 3.6. Antimicrobial Activity and Optimization of Culture Parameters

**Figure 7:**
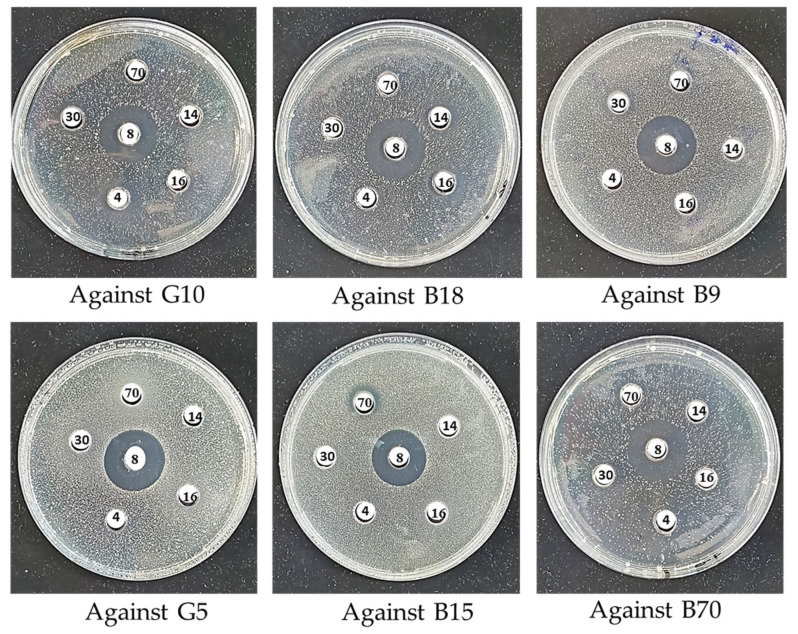
Agar well diffusion assay showing inhibition zones (clear halos) around wells containing thermophilic bacterial isolates. Six isolates (B16, B30, B70, B8, G14, and G4) were tested against six target isolates (G10, B18, B9, G5, B15, and B70). Wells are labeled according to isolate numbers (16, 30, 70, 8, 14, 4) on each Petri dish, which is labeled with the corresponding target isolate. The size of the clear halo around each well reflects the antimicrobial potency of the isolate. Inhibition zone diameters were measured to compare the effectiveness of the isolates.

The selection of producer isolates for antimicrobial assays was based on preliminary phenotypic screening (growth kinetics, pigment production, sporulation, and colony morphology) and phylogenetic diversity to ensure a broad taxonomic and ecological representation within the 30 isolates. This strategy aimed to identify isolates with a higher potential for secondary metabolite production, including antimicrobial compounds. For the target isolates, we focused on evaluating intra- and interspecific antimicrobial interactions within the thermophilic microbial community, as such interactions are critical for survival in extreme environments. The selected target isolates were closely related thermophiles from the same or similar thermal habitats, enabling us to simulate ecological pressures and assess selective activity against related competitors, which may indicate a competitive advantage in situ. Six thermophilic isolates (*G. thermoleovorans* B8, *G. kaustophilus* B70, *G. thermoleovorans* B30, *G. kaustophilus* B16, *G. stearothermophilus* G14, and *B. aydinogluensis* G4) were tested for antimicrobial activity against six related thermophilic targets (*G. kaustophilus* B15, *G. vulcani* B18, *G. kaustophilus* B70, *G. kaustophilus* B9, *G. kaustophilus* G10, and *B. aydinogluensis* G5) using the agar well diffusion method (Figure 7). The results revealed varying inhibition capacities among the tested isolates. The *G. thermoleovorans* B8 isolate exhibited the strongest and broadest activity, with inhibition zones reaching up to 24 mm, particularly against *G. kaustophilus* G10, *G. kaustophilus* B9, *G. vulcani* B18, and *B. aydinogluensis* G5. Moderate activity was observed in *G. kaustophilus* B16, with inhibition zones of 16 mm, while *G. kaustophilus* B70, *G. thermoleovorans* B30, *B. aydinogluensis* G4, and *G. stearothermophilus* G14 showed weaker inhibition (≤14 mm) or no observable effect (Table 3). Statistical analysis confirmed significant differences between the isolates (*p* < 0.001), especially in comparisons involving *G. thermoleovorans* B8 (Table 3; Supplementary Figure S1). Treatment of cell-free supernatants with proteinase K resulted in a complete loss of antimicrobial activity, as evidenced by the absence of inhibition zones in the agar well diffusion assay. In contrast, untreated supernatants maintained clear inhibitory effects against the indicator isolates. Moreover, the production of antibacterial peptides proved unstable during repeated assays, indicating sensitivity to environmental or physiological factors. The optimization of culture conditions identified soft agar MNA medium supplemented with 0.25% Gelrite (Serva) as the most effective for enhancing antimicrobial expression. The combination of agar and Gelrite was used to improve the texture, clarity, and consistency of the solid medium. Agar provides the typical solidification properties, while Gelrite enhances gel strength and stability, particularly under high-temperature conditions, and contributes to the clarity of the medium. This was crucial for improving both zone clarity and accuracy, especially for the cultivation of thermophilic isolates [67,68]. Maximum activity was observed after 24 h incubation at 60 °C. Following repeated subculturing and storage, all six antimicrobial-producing isolates exhibited a marked decline or complete loss of inhibitory activity. To address this, co-culture assays were performed using inactivated *E. faecium* supernatant. This treatment consistently restored detectable antimicrobial inhibition across all tested isolates.

## 4. Discussion

The unique ability of thermophilic microorganisms to survive in extreme environments continues to offer valuable prospects for biotechnological advancement, particularly in the production of AMPs. Their heat-stable biomolecules, adapted to high temperatures and fluctuating physicochemical conditions, are of particular interest for applications in biomedicine, agriculture, and industry [69,70].

The current study investigated thermophilic bacterial isolates from two Algerian geothermal springs, using a polyphasic approach combining phenotypic, proteomic, and molecular tools to explore taxonomic diversity and functional traits related to antimicrobial potential. Similar approaches have been successfully employed in studies characterizing extremophiles from geothermal environments, where species such as *Geobacillus*, *Aneurinibacillus*, and *Brevibacillus* have shown promising antimicrobial and enzymatic properties [71,72,73,74].

The use of MALDI-TOF MS and 16S rRNA sequencing provided complementary identification tools [75]. As previously reported by Kopcakova et al. and Rahi et al. [27,76], MALDI-TOF MS proved effective for rapid preliminary identification, while 16S rRNA gene sequencing provided higher taxonomic resolution, particularly for differentiating closely related species such as *B. licheniformis* and *B. paralicheniformis*. The prevalence of *G. kaustophilus* at Hammam Bouhadjar may be attributed to the spring’s near-neutral pH (6.7–7) and its rich mineral composition, particularly in sulfate, calcium, and bicarbonate ions [77,78]. These geochemical factors align with the ecological preferences of *Geobacillus* species, which are commonly found in similar environments [79]. Specifically, *G. kaustophilus* is well-adapted to such conditions due to its ability to form spores, its osmoregulatory mechanisms, and its thermostable enzymes, which confer resilience to both thermal and ionic stress [80,81]. Water pH plays a crucial role in shaping microbial diversity in hot springs, as noted by Chan et al. [82]. Similarly, Rowe et al. [83] also found that pH is a primary driver of microbial diversity in geothermal environments, with higher water pH correlating with increased alpha and beta diversity. Additionally, higher concentrations of dissolved heavy metals were associated with reduced microbial diversity in both water and sediment, reinforcing the role of environmental factors in shaping microbial communities [83].

The application of rep-PCR fingerprinting methods ((GTG)_5_-PCR, BOX-PCR, and ERIC-PCR) revealed substantial genomic diversity, reflecting the genetic complexity reported in other thermophilic bacterial communities [29,44]. Comparatively, BOX-PCR offered the highest discriminatory power, especially at the strain level, in line with results from Efe and Ahmet and Wani et al. [84,85]. ERIC-PCR showed more limited resolution, consistent with observations by Savas et al. [86] and Oztas Gulmus and Gormez [87], but remained useful for capturing broader taxonomic relationships [86,87].

The results from fingerprinting methods and 16S rRNA gene sequencing were highly consistent. Fingerprinting methods, particularly (GTG)_5_-PCR, revealed significant genetic diversity and clustered isolates *from G. kaustophilus* and *B. licheniformis* in accordance with the phylogenetic relationships identified through 16S rRNA sequencing [88]. While fingerprinting provided detailed genetic profiles, 16S rRNA sequencing confirmed species-level identification and established clear phylogenetic relationships, demonstrating the congruence between both approaches in identifying and classifying thermophilic isolates [89].

The lack of strict correlation between genetic clustering and geographic origin suggests that environmental pressures, such as temperature or mineral content, exert stronger selective forces than geographic distance alone, corroborating the conclusions of Najar et al. and Wang et al. [90,91], as well as those of Ulucay et al. [92] who found no significant difference between the groups or the bacteria regarding their geographic locations or optimal temperatures [92]. The presence of genetically distinct isolates (e.g., *G. kaustophilus* B14, *G. thermoleovorans* B30) within similar ecological settings further supports the notion of micro-niche adaptation and genomic plasticity among thermophiles [93,94].

Variation in antimicrobial activity among isolates, particularly within *Geobacillus* spp., is likely influenced by both genetic regulation and environmental factors. The complete loss of antimicrobial activity after proteinase K treatment confirms the proteinaceous nature of the compounds, consistent with bacteriocin characteristics. This finding aligns with previous reports showing that proteolytic enzymes such as proteinase K, pronase, and trypsin degrade bacteriocins [11,95,96]. Furthermore, prior studies have demonstrated that bacteriocin gene expression in thermophilic bacteria is tightly regulated and dependent on environmental conditions [97,98,99,100]. Additionally, resistance in target isolates may also reduce observable activity, due to receptor modifications such as those affecting Man-PTS systems [101].

Comparative studies further confirm that even closely related thermophilic strains can exhibit markedly different antimicrobial profiles. While *Geobacillus* sp. ZGt-1 has demonstrated broad-spectrum activity [102], others under similar conditions show minimal inhibition, likely due to silent gene clusters or post-translational repression [103].

Notably, bacteriocin production in our isolates showed instability after repeated subculturing and long-term storage. This phenomenon has been previously reported, including Merzoug et al. [38], who suggested that laboratory domestication under optimal growth conditions may suppress stress-induced biosynthetic pathways. To overcome this, we implemented optimization strategies involving modified culture media and co-culture systems, which successfully restored the antimicrobial activity, in line with findings by Bertrand et al. [104] and Hockett and Baltrus [105].

In particular, co-cultivation with inactivated *E. faecium* supernatant effectively reactivated antibacterial peptide production across the tested isolates. This approach simulates natural microbial interactions and has been shown to stimulate secondary metabolite biosynthesis through stress-response and quorum-sensing mechanisms [106]. Similar results have been reported in both mesophilic and thermophilic systems [107,108], as well as in actinomycetes exposed to inactivated *Staphylococcus aureus* and *Escherichia coli*, which led to enhanced antimicrobial activity [60]. Recent studies also demonstrated increased bacteriocin output in *Bacillus velezensis* and *Lactiplantibacillus paraplantarum* co-cultured with yeast [109]. These techniques represent promising, scalable alternatives to genetic engineering for enhancing antimicrobial metabolite production, particularly in industrial fermentation and biopreservation contexts [110,111].

## 5. Conclusions

This study characterized 30 thermophilic bacterial isolates from Algerian hot springs, with *Geobacillus* emerging as the dominant genus. Polyphasic identification using MALDI-TOF MS and 16S rRNA sequencing confirmed *Geobacillus* as the dominant genus. These thermophilic isolates offer promising applications in agriculture, pharmaceuticals, and biopreservation due to their production of thermostable antimicrobial compounds. Their resilience and inducible activity make them strong candidates for use in high-temperature industrial processes. Future research should prioritize genome sequencing of active isolates, such as *G. thermoleovorans* B8, to identify antimicrobial gene clusters and regulatory elements. Complementary strategies, including metabolomic profiling, fermentation optimization, and CRISPR-Cas-based functional analysis, could further enhance production and broaden their use against resistant pathogens. Thermophilic bacteria thus represent a sustainable solution to current antimicrobial challenges.

## Figures and Tables

**Table 1:** Primers synthesized for cloning experiments carried out in this study.

Category	Target Gene	Forward Primer (5′ → 3′)	Reverse Primer (5′ → 3′)	Amplicon Size (bp)	Tm (°C)	Reference
Molecular identification	16S rRNA	AGAGTTTGATCCTGGCTCAG	ACGGCTACCTTGTTACGACTT	1485	56	[44,45]
Genotyping	ERIC	ATGTAAGCTCCTGGGGATTCAC	AAGTAAGTGACTGGGGTGAGCG	/	52	[46]
	(GTG)_5_	GTGGTGGTGGTGGTG	/	/	45	[47]
	BOX	CTACGGCAAGGCGACGCTGACG	/	/	53	[47]

**Table 2:** Proteomic (MALDI-TOF MS) and molecular (16S rRNA) characterization of thermophilic bacterial isolates.

Isolate Code	Proteomic Identification (MALDI-TOF MS)	MALDI-TOF Score	Molecular Identification (16S rRNA Gene Sequencing)	Similarity Rate (%) *	Closest Phylogenetic Relative (GenBank Accession Number) **
G4	*Brevibacillus aydinogluensis*	2.23	*Brevibacillus aydinogluensis*	99.32%	*Brevibacillus aydinogluensis* (NR_117986.1)
G5	*Brevibacillus aydinogluensis*	2.28	*Brevibacillus aydinogluensis*	99.32%	*Brevibacillus aydinogluensis* (NR_117986.1)
G6	*Brevibacillus aydinogluensis*	2.24	*Brevibacillus aydinogluensis*	98.64%	*Brevibacillus aydinogluensis* (NR_117986.1)
G10	*Geobacillus kaustophilus*	2.32	*Geobacillus kaustophilus*	98.49%	*Geobacillus kaustophilus* (NR_115285.2)
G11	*Geobacillus kaustophilus*	1.98	*Geobacillus proteiniphilus*	100%	*Geobacillus proteiniphilus* (NR_178413.1)
G13	*Geobacillus stearothermophilus*	2.07	*Geobacillus stearothermophilus*	99.40%	*Geobacillus stearothermophilus* (NR_115284.2)
G14	*Geobacillus stearothermophilus*	2.1	*Geobacillus stearothermophilus*	99.64%	*Geobacillus stearothermophilus* (NR_115284.2)
G30	*Bacillus licheniformis*	2.01	*Bacillus licheniformis*	99.45%	*Bacillus licheniformis* (NR_118996.1)
G50	*Brevibacillus aydinogluensis*	1.85	*Brevibacillus thermoruber*	99.74%	*Brevibacillus thermoruber* (NR_112213.1)
G60	*Aneurinibacillus thermoaerophilus*	2.31	*Aneurinibacillus thermoaerophilus*	99.59%	*Aneurinibacillus thermoaerophilus* (NR_112216.1)
B3	*Bacillus licheniformis*	1.93	*Bacillus paralicheniformis*	100%	*Bacillus paralicheniformis* (NR_137421.1)
B5	*Geobacillus kaustophilus*	2.14	*Geobacillus kaustophilus*	99.75%	*Geobacillus kaustophilus* (NR_114089.1)
B6	*Geobacillus kaustophilus*	2.23	*Geobacillus kaustophilus*	99.87%	*Geobacillus kaustophilus* (NR_114089.1)
B7	*Geobacillus kaustophilus*	2.13	*Geobacillus kaustophilus*	99.80%	*Geobacillus kaustophilus* (NR_114089.1)
B8	*Geobacillus thermoleovorans*	2.24	*Geobacillus thermoleovorans*	99.62%	*Geobacillus thermoleovorans* (NR_115286.2)
B9	*Geobacillus kaustophilus*	2.01	*Geobacillus kaustophilus*	99.49%	*Geobacillus kaustophilus* (NR_114089.1)
B10	*Geobacillus kaustophilus*	2.37	*Geobacillus kaustophilus*	99.86%	*Geobacillus kaustophilus* (NR_115285.2)
B11	*Geobacillus kaustophilus*	1.97	*Geobacillus proteiniphilus*	100%	*Geobacillus proteiniphilus* (NR_178413.1)
B14	*Geobacillus kaustophilus*	2.04	*Geobacillus kaustophilus*	99.32%	*Geobacillus kaustophilus* (NR_115285.2)
B15	*Geobacillus kaustophilus*	2.23	*Geobacillus kaustophilus*	97.96%	*Geobacillus kaustophilus* (NR_114089.1)
B16	*Geobacillus kaustophilus*	2.14	*Geobacillus kaustophilus*	97.32%	*Geobacillus kaustophilus* (NR_114089.1)
B17	*Geobacillus kaustophilus*	1.83	*Geobacillus proteiniphilus*	100%	*Geobacillus proteiniphilus* (NR_178413.1)
B18	*Geobacillus jurassicus*	1.98	*Geobacillus vulcani*	99.55%	*Geobacillus vulcani* (NR_025426.1)
B20	*Geobacillus kaustophilus*	2.18	*Geobacillus proteiniphilus*	99.88%	*Geobacillus proteiniphilus* (NR_178413.1)
B21	*Geobacillus kaustophilus*	2.17	*Geobacillus kaustophilus*	99.40%	*Geobacillus kaustophilus* (NR_114089.1)
B22	*Geobacillus kaustophilus*	2.27	*Geobacillus kaustophilus*	98.92%	*Geobacillus kaustophilus* (NR_114089.1)
B30	*Geobacillus jurassicus*	1.77	*Geobacillus thermoleovorans*	100%	*Geobacillus thermoleovorans* (NR_115286.2)
B40	*Aeribacillus pallidus*	2.15	*Aeribacillus pallidus*	99.68%	*Aeribacillus pallidus* (NR_026515.1)
B50	*Aeribacillus pallidus*	2.39	*Aeribacillus pallidus*	99.84%	*Aeribacillus pallidus* (NR_026515.1)
B70	*Geobacillus kaustophilus*	2.17	*Geobacillus kaustophilus*	99.92%	*Geobacillus kaustophilus* (NR_115285.2)

* Degree of similarity to the 16S rRNA gene sequence of the closest phylogenetic match. ** The average cleaned and aligned consensus length for all 30 thermophilic isolates compared with GenBank was 800 ± 20 bp.

**Table 3:** Antimicrobial activity of six selected thermophilic isolates (zone of growth inhibition in mm, including the 6 mm diameter of the well).

Target Isolates	Inhibitory Isolates					
	G4	B8	G14	B16	B30	B70
G10	14	23	-	11	11	-
B18	-	24	14	16	-	-
B9	-	23	-	-	11	-
G5	-	23.5	-	-	-	-
B15	-	22.5	-	-	-	13
B70	-	22.5	-	-	12	11

(-) indicates no inhibition zone.

## Data Availability

The original contributions presented in this study are included in the article/Supplementary Material. Further inquiries can be directed to the corresponding authors.

## Supplementary Materials

The following supporting information can be downloaded at: https://www.mdpi.com/article/10.3390/microorganisms13061425/s1, Figure S1: Screening of antimicrobial activity of six thermophilic producer isolates (B16, B30, B70, B8, G14, and G4) against related thermophilic indicator isolates (G15, B18, B70, B9, G10, and G5) using the well diffusion assay, based on inhibition zone diameters.
