# Supplementary material for: Taxonomic Diversity and Antimicrobial Potential of Thermophilic Bacteria from Two Extreme Algerian Hot Springs
Source: Microorganisms. 2025 Jun 19;13(6):1425. doi: 10.3390/microorganisms13061425 (PMC12196180; doi:10.3390/microorganisms13061425)
Supplement: Supplementary file 1 [file microorganisms-13-01425-s001.zip › microorganisms-3686735-supplementary.pdf]

Supplementary Information

Taxonomic Diversity and Antimicrobial Potential of Thermophilic Bacteria from Two Extreme Algerian Hot Springs

Marwa Aireche [1], Mohamed Merzoug [1,*], Amaria Ilhem Hammadi [1], Zohra Yasmine Zater [2], Keltoum Bendida [1], Chaimaa Naila Brakna [1], Meryem Berrazeg [3], Ahmed Yassine Aireche [4], Yasmine Saidi [1], Svetoslav Dimitrov Todorov [5,6,*], Dallel Arabet [7] and Djamal Saidi [1]

[1] Higher School of Biological Sciences of Oran, BP 1042 Saim Mohamed, Cité Emir Abdelkader (EX-INESSMO), Oran 31000, Algeria; marwa.ar231@gmail.com (M.A.); hammadiamaria267@gmail.com (A.I.H.); keltoumbendida2001@gmail.com (K.B.); chaimaa1012@gmail.com (C.N.B.); yasmine.saidi@gmail.com (Y.S.); djamsaidi@gmail.com (D.S.)

[2] Laboratory of Microorganisms Biology and Biotechnology, University of Oran 1 Ahmed Ben Bella, Oran 31000, Algeria; yassminezater93@gmail.com

[3] Biotechnology Laboratory for Food and Energy Security, University of Oran 1 Ahmed Ben Bella, Oran 31000, Algeria; berrazegmeryem@gmail.com

[4] Computer Science Department, University of Abu Bekr Belkaid, Tlemcen 13000, Algeria; yassinetakiko@gmail.com

[5] ProBacLab, Laboratório de Microbiologia de Alimentos, Departamento de Alimentos e Nutrição Experimental, Food Research Center, Faculdade de Ciências Farmacêuticas, Universidade de São Paulo, São Paulo 05508-000, SP, Brazil

[6] Department of General Hygiene, I.M. Sechenov First Moscow State Medical University, Trubetskaya St., Bldg. 8/2, Moscow 119435, Russia

[7] Laboratory of Microbiological Engineering and Applications, Faculty of Natural and Life Sciences, University of Constantine 1, Constantine 25000, Algeria; dallelarabet@yahoo.fr

* Correspondence: merzoug.mohamed1@yahoo.fr (M.M.); slavi310570@abv.bg (S.D.T.)

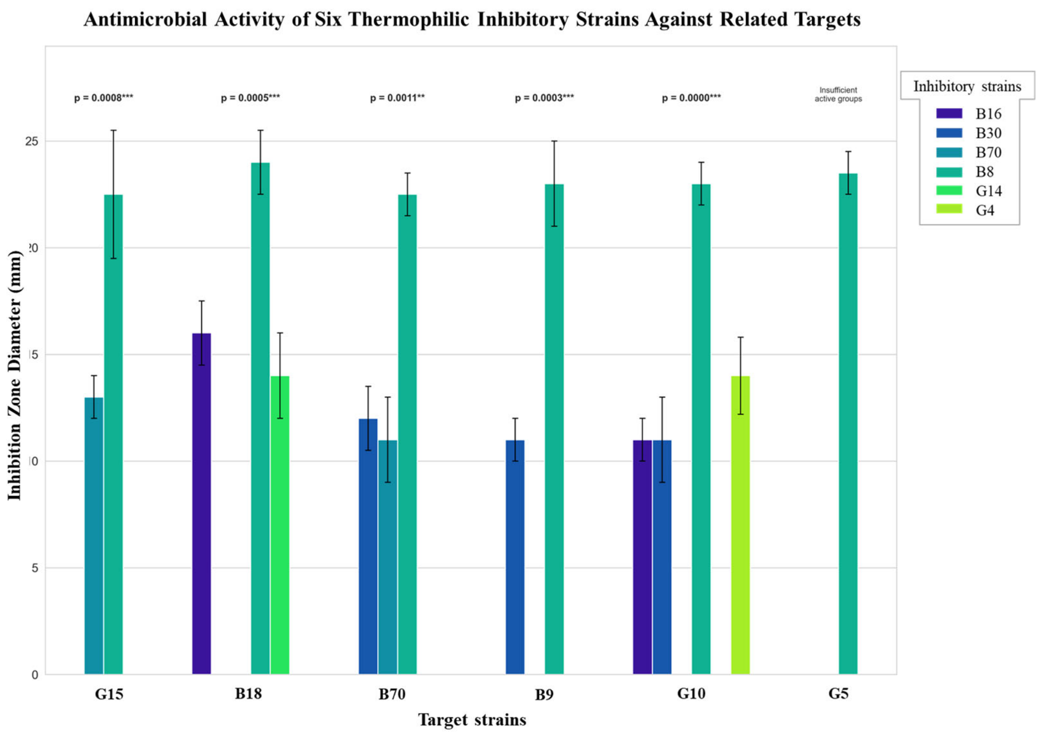

Supplementary Figure S1. Screening of antimicrobial activity of six thermophilic producer isolates (B16, B30, B70, B8, G14, and G4) against related thermophilic indicator isolates (G15, B18, B70, B9, G10, and G5) using the well diffusion assay, based on inhibition zone diameters.
